# The role of serotonin 1B in the representation of outcomes

**DOI:** 10.1038/s41598-019-38938-4

**Published:** 2019-02-21

**Authors:** Laura Corbit, Michael Kendig, Caroline Moul

**Affiliations:** 1 School of Psychology, University of Sydney, Sydney, Australia; 2 Department of Psychology, University of Toronto, Toronto, Canada; 3 School of Medical Sciences, University of New South Wales, Sydney, Australia

## Abstract

Disrupted serotonin neurotransmission has been implicated in the etiology of psychopathic traits. Empirical research has found that people with high levels of psychopathic traits have a deficit in reinforcement learning that is thought to be linked with amygdala dysfunction. Altered serotonin neurotransmission provides a plausible explanation for amygdala dysfunction in psychopathic traits and recent research suggests that this may be associated with serotonin 1B (5-HT_1B_) receptor function. This research used an animal model to test the hypothesis that 5-HT_1B_ receptors are involved in the encoding of the specific features of reinforcing outcomes. An outcome devaluation task was used to test the effect of the systemic administration of a selective 5-HT_1B_ receptor agonist administered before encoding of “action-outcome” associations. Results showed that while administration of a 5-HT_1B_ receptor agonist allowed rats to acquire instrumental responding for food, when the content of that learning was further probed using an outcome devaluation task, performance differed from controls. 5-HT_1B_ agonism impaired learning about the specific sensory qualities of food rewards associated with distinct instrumental responses, required to direct choice performance when the value of one outcome changed. These findings suggest a role for 5-HT_1B_ receptor function in the encoding of the specific features of reinforcing outcomes.

## Introduction

Individuals with high levels of psychopathic personality traits (PT) are characterized by low levels of empathy, diminished feelings of shame and guilt, and limited prosocial emotions. High levels of PT are a risk factor for negative outcomes that endure across the life course (e.g. criminal behaviour, relationship difficulties, and a diagnosis of antisocial personality disorder^1^). The negative outcomes associated with high levels of PT in adults are also relevant for children and adolescents with high levels of the developmental analogue of PT (callous-unemotional traits)^2^. A current focus of research is to elucidate the processes underlying the development of PT. One mechanism that is implicated is associative learning.

Research clearly shows that individuals with high levels of PT, both with and without concurrent antisocial behaviour problems, have a deficit in reinforcement learning^3–6^. A variety of different tasks have been used to illustrate the difficulties with reinforcement learning exhibited by people with PT and, over the course of several decades, research has narrowed in on the specific nature of this problem. One of the most informative tasks has been the response-reversal learning task. These tasks typically begin with an acquisition phase in which stimuli are presented in pairs; one stimulus in each pair leads to reward when selected (e.g., win 100 points), and the other leads to a loss (e.g., lose 100 points). As such, the subject learns to respond to some stimuli and avoid others depending on whether they are rewarding or punishing. In the second phase of the task, stimulus-outcome contingencies are changed such that previously rewarded stimuli now lead to loss and vice versa. Thus, the subject will perform better on each trial if they stop selecting the stimulus that was previously rewarding and instead selects the one that they had previously learnt to avoid. Studies have demonstrated that while people with PT generally show no deficits in acquisition (they initially learn to select the rewarding stimuli and avoid the punishing stimuli at the same rate as people without PT), they are slower to reverse their pattern of stimulus selection^5,6^.

Potential explanations for this pattern of behaviour include an insensitivity to punishing outcomes^7,8^, or reward dominance (behaviour driven more by seeking reward than by avoiding punishment)^9^. However, further research demonstrates that the reinforcement learning deficits shown by people with PT may be independent from motivational properties. Blair, Morton *et al*.^10^ conducted a Differential Reward/Punishment Learning Task in which 2 of 10 possible pictorial stimuli were displayed together on a screen at a time. The subject was instructed to select one of the two stimuli. Half of the stimuli led to a win while the other half led to a loss. Critically, each of the 5 winning stimuli were associated with a different number of points (+1600, +800, +400, +200, +100) which was mirrored by the 5 stimuli that led to loss. During testing, all stimuli were presented with one another such that, throughout the experiment, trials could either include two stimuli that led to rewards of different values, two stimuli that led to losses of different values, or one winning stimulus and one losing stimulus. Thus, the valence (win or loss) associated with a stimulus was not sufficient for optimizing choice. Instead, participants had to recall the specific points associated with each stimulus and judge the value of those points relative to the other stimulus present on that trial (e.g., +400 is better than +200, but worse than +800). Participants with PT performed worse than controls in this task. Their ability to associate a specific value with an arbitrary stimulus and judge this value relative to alternatives to optimize performance was impaired relative to the controls.

Evidence from the human research literature indicates that serotonin neurotransmission plays a role in both psychopathic traits^11–14^ and in associative learning in humans^15,16^. However, serotonin has multiple classes of receptors with distinct and widespread functions^17^. Importantly, recent evidence specifically implicates the serotonin 1B (5-HT_1B_) receptor in the etiology of PT. Moul *et al*.^14^ found an association between a functional genetic polymorphism (rs11568817) of the serotonin 1B receptor gene (*HTR1B*) and callous-unemotional traits (the childhood analogue of PT) in a sample of boys with antisocial behaviour problems. This polymorphism is of specific interest as it is known to have a functional role in the serotonin system. The minor allele contributes to the creation of a transcription factor binding site that results in a 2.3-fold increase in gene expression^18^. Thus, this result^14^ would suggest that increased expression of *HTR1B*, linked to the presence of the minor allele of rs11568817, is associated with PT. Indeed, further research conducted by this group^13^ found that methylation of the promoter region of *HTR1B* was also associated with PT in boys with antisocial behaviour problems.

Methylation is a dynamic process that can impact gene expression by influencing the degree to which DNA is transcribed. Typically, increased methylation is associated with reduced gene expression. Moul *et al*. (2015) found that PT in boys with antisocial behaviour problems was negatively associated with methylation at two locations in the promoter region of *HTR1B*: the less methylation in these locations the higher the PT. Together, the genetic and epigenetic results suggest that increased expression of *HTR1B* may be relevant for the development of PT. Support for this hypothesis comes from the animal literature; it has been demonstrated that increased expression of *HTR1B* in rats decreases fear-potentiated startle^19^ a response that is also reliably reduced in people with PT^8^.

The 5-HT_1B_ receptor functions predominantly as a terminal autoreceptor, inhibiting serotonin release at serotonin terminals^20,21^. Thus, 5-HT_1B_ terminal autoreceptors located on axons originating in the midbrain raphe nuclei can act as part of a negative feedback system, changing the rate of serotonin release in response to localized fluctuations in extracellular serotonin. Interestingly, a negative relationship between PT and serum serotonin levels was found in the *HTR1B* methylation research sample^13^ which supports a potential functional role of diminished *HTR1B* methylation on serotonin neurotransmission.

The overall aim of the following study was to link altered reinforcement learning to serotonergic activity and test the hypothesis that 5-HT_1B_ receptors are involved in the encoding of the specific features of reinforcing outcomes. It was hypothesized that increased *HTR1B* expression, as found to be associated with PT, could be mimicked via the use of a selective 5-HT_1B_ agonist; the agonist would result in a downregulation of serotonin-induced activation of forebrain afferents important for detailed outcome encoding. Before conducting more in-depth studies to test the role of specific brain regions, it was first necessary to use animal models to test the basic premise: that reinforcement learning processes are influenced by the activation of 5-HT_1B_ receptors during encoding.

To this end, hungry animals were trained to make two instrumental responses (left and right lever presses) for distinct food outcomes (grain pellets and 20% sucrose solution). To assess the involvement of 5-HT_1B_ in the *encoding* of these specific ‘action-outcome’ associations, half of the animals were given systemic administration of the 5-HT_1B_ receptor agonist CP94253 prior to daily training sessions in which animals earned both food rewards. The remaining animals were given vehicle injections. All animals were drug-free for the subsequent test phase, which assessed animals’ sensitivity to outcome devaluation. In these tests animals first received free access to either pellets or sucrose solution, immediately prior to a brief test in the training chambers where responding on both levers was measured. Animals typically selectively reduce responding on the lever earning the devalued outcome. Such performance reflects intact encoding of the specific features of each outcome and association of these outcomes with the distinct responses during earlier training which permits value-based choice at test. If specific action-outcome encoding is disrupted during training, as hypothesized to occur following 5-HT_1B_ receptor agonism, this should be reflected in reduced sensitivity to outcome devaluation, as measured by a failure to selectively reduce responding on the lever associated with the devalued outcome.

## Methods

All experimental procedures were carried out in accordance with the recommendations of the Australian code for the care and use of animals for scientific purposes 8th edition (2013) and were approved by the Animal Ethics Committee at the University of Sydney (Protocol Number 2016/1080).

Twenty-four experimentally naive male adult hooded Wistar rats (University of Adelaide) were used. They were group-housed 4 per cage in a temperature- and humidity-controlled colony room maintained on a reverse dark:light cycle (lights off 0900–2100 h) and had free access to tap water throughout all experimental procedures. Chow access was initially *ad-libitum* during elevated plus maze testing but was then restricted to 12 g/rat/day throughout instrumental training and testing.

### Drug administration

The 5-HT_1B_ agonist CP94253 (Sigma, USA) was dissolved in distilled water (5 ml/kg) and was administered i.p. at a dosage of 5 mg/kg. This dose was based on previous studies demonstrating that this dose had no effect on milk or sucrose self-administration or locomotor activity, but was effective in reducing cocaine self-administration and reinstatement of cocaine-seeking^22,23^.

### Elevated Plus Maze test

The acute effects of 5-HT_1B_ agonism were first assessed in the Elevated Plus Maze^24^, a well-validated measure of anxiety in rodents. Previous research has demonstrated an anxiogenic effect of CP94253^25^ and so this test was included as a positive control for behavioural effects of the selected drug dose. The apparatus was a plus-shaped maze elevated 1 m above the ground, with two closed arms surrounded by high (40 cm) Perspex walls, and two open arms. Animals are placed in the center of the maze and allowed to explore for 5-min; more time spent exploring the open arms is taken to indicate lower levels of anxiety.

### Instrumental training

In preparation for instrumental training a restricted feeding schedule was introduced wherein animals were fed a ration of 12 g chow per rat, per day. Twelve identical operant chambers (Med-Associates, USA) were used for training and test procedures. The floor consisted of steel bars with top and sidewalls made of clear Plexiglas and end walls made of aluminum. Two retractable levers were located on one side of the chamber with a recessed magazine centered between them. A 3-W, 24-V house-light provided illumination. The left and right levers were assigned to earn grain pellets (45 mg, grain-based formula, Bioserv, USA) and 20% sucrose solution (w/vol; ~0.2 ml per reward) in a counterbalanced fashion. Devaluation pre-feeding was conducted in individual plastic cages with open wire tops. Rats were habituated to these cages prior to testing.

### Procedure

Instrumental training began with a single 40-min session of magazine training in which 15 aliquots of 20% maltodextrin solution (0.2 ml) were delivered to the magazine. Next, all rats were trained to make both left and right instrumental responses with each lever-press reinforced with 0.1 ml of a 20% maltodextrin solution. There were two of these sessions to ensure that all rats had the opportunity to learn to respond and that any drug effects would not interfere with initial instrumental acquisition but would instead be restricted to the period of training when rats could encode the specific features of the sucrose and pellet rewards subsequently used as rewards for left and right lever press responses. Both left and right levers were trained within a single session: the left (or the right) lever was inserted into the chamber until the animal earned five outcomes. At this point the lever retracted and, 10 s later, the right (or the left) lever was inserted until five of the other outcome were earned. This process repeated until animals earned 60 total rewards, or 60 min elapsed.

Eighteen rats successfully acquired both left and right lever press responses for 20% maltodextrin. Thirty minutes prior to each of five subsequent training sessions, nine of these rats received CP94253 (5 mg/kg, i.p.) and the other 9 received vehicle (distilled water 1 ml/kg). Responding during these training sessions was reinforced on a random-ratio 5 schedule, meaning one outcome was delivered after 5 lever-press responses, on average.

### Devaluation testing

Two devaluation tests were held drug-free, ensuring that differences in responding were due to the effects of 5HT_1B_ receptor agonism on the associations learned during prior training. Devaluation was achieved by specific satiety. Rats were tested twice on separate days with one session of retraining and drug treatment between the two tests. On each test day, rats were placed in individual feeding cages and given free access to either pellets or sucrose solution for 1-h. They were then transferred immediately to the training chambers for a 5-min extinction test in which both left and right levers were presented, but presses were not reinforced. A second test was identical to the first except that rats were pre-fed the opposite food (e.g., rats that were pre-fed pellets in test 1, were pre-fed sucrose in test two, and vice versa). Sensitivity to devaluation is reflected in lower responding on the lever earning the devalued outcome relative to the lever earning the non-devalued outcome.

### Consumption Test

Animals were given a single consumption test to confirm the efficacy of the devaluation treatment. In this test half of the rats in each group were pre-fed pellets and the other half were pre-fed sucrose for 1-hr in the individual pre-feeding cages used for devaluation tests. These foods were then removed and all animals were given a fresh dish with 20 g of pellets. These dishes were removed 10 minutes later and consumption recorded. Lower pellet consumption following prior consumption of pellets relative to prior consumption of sucrose would confirm that specific-satiety was intact, and thus that devaluation treatment was effective.

### Reinforced Test

A reinforced test was conducted five days later. Half of the animals in each group were pre-fed pellets and the other half were pre-fed sucrose for 1-h. They were then transferred immediately to the training chambers where both levers remained present throughout a 20-min test. The first lever press on either lever was reinforced; thereafter, responding was reinforced using independent reinforcement schedules that ascended from random-ratio 3 (RR3) for rewards 2–5, to RR5 for rewards 6–10, and RR10 for subsequent rewards. The test lasted 20 minutes.

## Results

### Elevated plus maze

**Figure 1:**
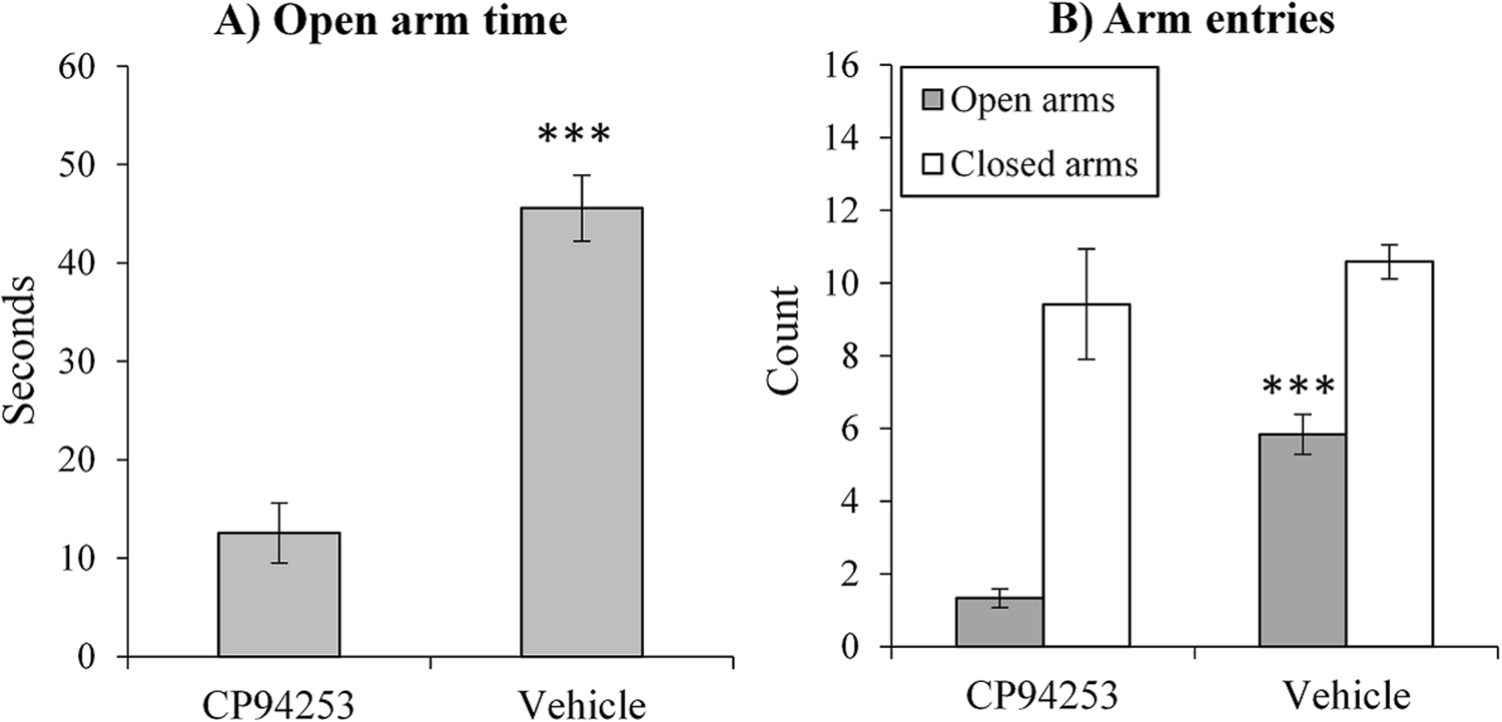
Elevated plus maze. CP94253 treatment significantly decreased time spent on open arms in the elevated plus maze, (Panel A; ***p < 0.001). CP94253 treatment also decreased the number of entries into the open, but not closed arms (Panel B; ***p < 0.001) indicating that acute CP94253 administration increased anxiety-like behaviour and suggesting that results were unlikely to be secondary to locomotor impairment.

As shown in Fig. 1A, CP94253 administration (versus vehicle) 30-min prior to test significantly reduced time spent on the open arms (*F*(1, 22) = 53.21, *p* < 0.001) and reduced the number of entries made into the open arms of the maze (Fig. 1B; *F*(1, 22) = 55.30, *p* < 0.001). By contrast, there was no significant effect on the number of entries into closed arms (*F* < 1) suggesting that CP94253 did not simply reduce exploration or locomotor activity. Together, this pattern of results indicated that 5-HT_1B_ agonism was anxiogenic.

### Instrumental training

**Figure 2:**
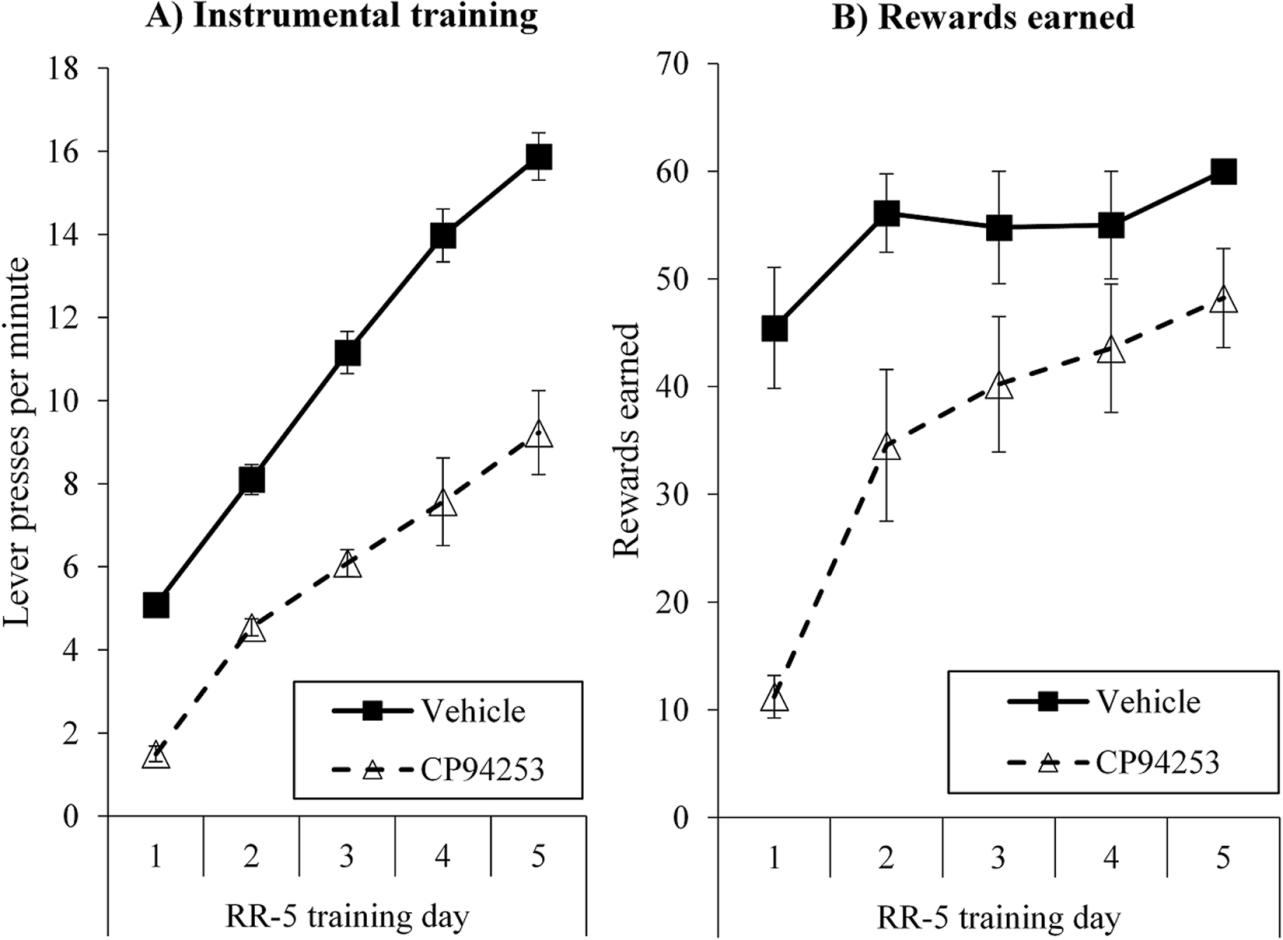
Instrumental training. Despite lower response rates during training (**A**) the CP94253 group still increased responding across days and earned a majority of the available outcomes in training sessions (**B**).

Results from instrumental training are shown in Fig. 2A. During the two days of instrumental pre-training when responding was reinforced with maltodextrin solution and no drug treatment given, response rates (lever presses/minute) for CP94253 and Vehicle groups were 1.21 ± 0.19 (SEM) and 1.15 ± 0.17 on day 1, and 2.58 ± 0.21 and 2.34 ± 0.36 on day 2. During the subsequent 5-days of instrumental training with sessions conducted following CP94253 or vehicle treatment, analyses indicated that response rates increased significantly across the 5 days of instrumental training (linear trend: *F*(1, 16) = 43.89, *p* < 0.001) and that this increase did not interact with group (linear interaction trend: *F*(1, 16) = 1.67, *p* = 0.21). Averaged over the 5 days of training, CP94253-treated rats responded at a significantly lower rate than vehicle-treated rats (main effect of group (*F*(1, 16) = 16.14, *p* = 0.001). Figure 2B shows that despite lower rates of responding, CP94253-treated rats still earned the majority of the 60 available outcomes in each daily training session (mean = 48 on day 5 of training for CP94253 group, versus 60 for vehicle group) and thus had the opportunity to learn about specific action-outcome associations.

### Devaluation tests

**Figure 3:**
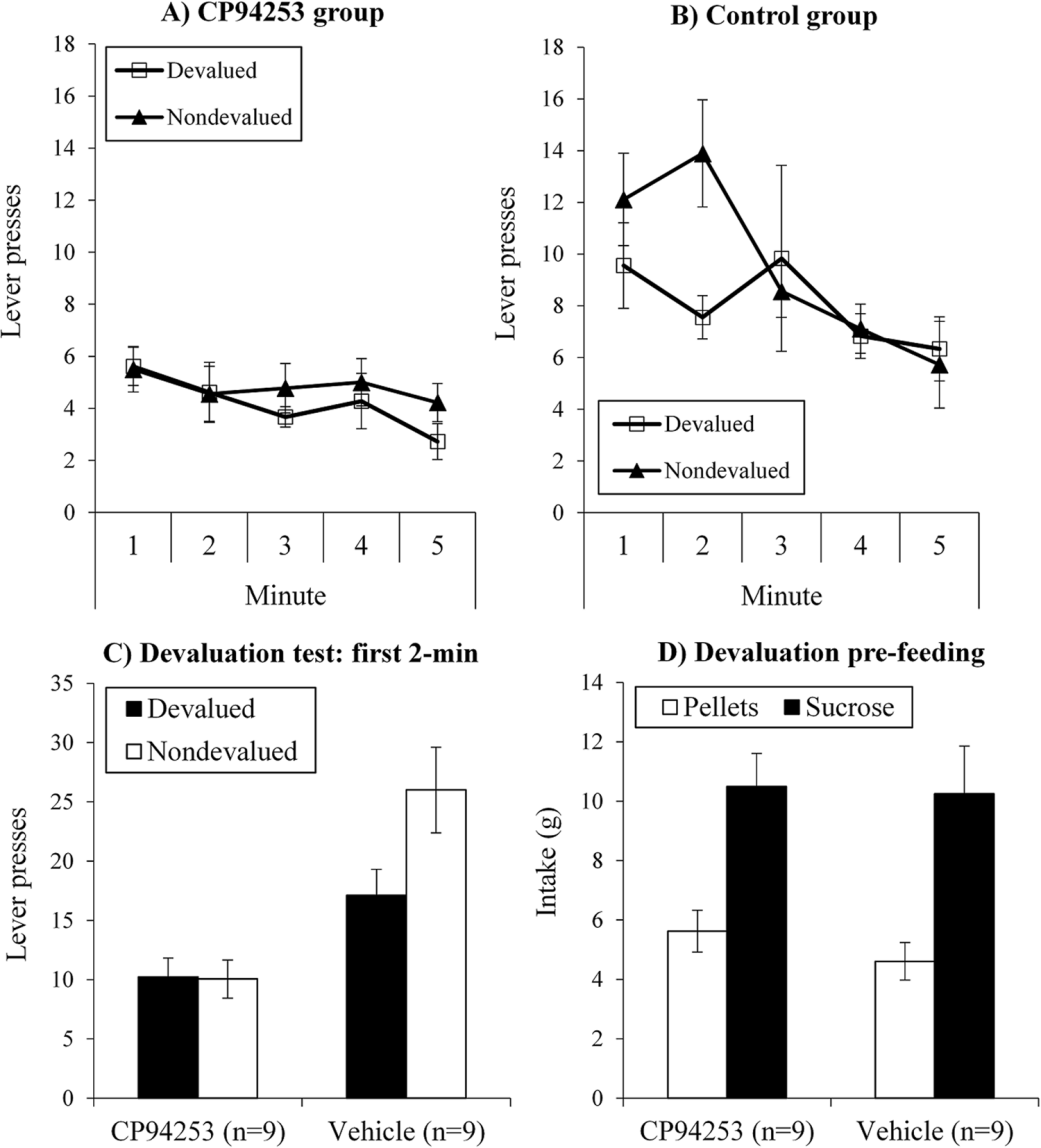
Devaluation test results. After 1-hr pre-feeding of one outcome, animals received a 5-min extinction test assessing responding on the levers that previously earned the now devalued and non-devalued outcomes. Analyses suggested insensitivity to devaluation treatment in CP94253-treated rats (Panel A) relative to the Control group (Panel B). (**C**) Responding in CP94253 and vehicle groups in the first 2 minutes of the extinction test. (**D**) Consumption (g) of the pre-fed outcome.

In the key test phase animals were placed in the training chambers for 5-min with both levers available and were free to respond but no rewards were delivered. Importantly, no drug was administered during devaluation tests, meaning differences in responding could only be attributed to associations formed during previous training. Figure 3A,B show bin data from the 5-min extinction test for CP94253 and vehicle groups. Data from the two tests are presented collapsed across outcome type because the pattern of responding was the same regardless of whether pellets or sucrose was the devalued outcome. Data split according to outcome are shown in Supplemental Fig. 1. A 2 × (2) × (5) ANOVA (group × [lever] × [minute]) indicated a significant 3-way interaction between group, lever, and minute (*F*(1, 16) = 6.51, *p* < 0.021) suggesting that sensitivity to devaluation varied between groups over the course of the test. In addition, this analysis found a significant overall devaluation effect (main effect of ‘lever’: *F*(1, 16) = 5.97, *p* = 0.027), that responding declined significantly over time (linear trend for ‘minute’: *F*(1, 16) = 21.84, *p* < 0.001), and was significantly lower in CP94253 animals, on average (group main effect: *F*(1, 16) = 12.45, *p* = 0.003).

Figure 3 and the 3-way interaction reported above suggested a group difference in sensitivity to devaluation in the early portion of the test. A 2 × (2) ANOVA applied to responding in the first 2-min of the test showed a significant group × [lever] interaction (*F*(1, 16) = 5.74, *p* = 0.029), a significant overall effect of lever (*F*(1, 16) = 5.32, *p* = 0.035) and a significant main effect of group (*F*(1, 16) = 16.33, *p* < 0.001). These results, shown in Fig. 3C and consistent with performance in the test overall, indicated that the group treated with CP94253 showed an inability to selectively direct responding toward the lever earning the nondevalued outcome. Tests of simple effects confirmed no effect of devaluation in the CP94253-treated group (*F*(1, 16) = 0.004; *p* = 0.95) in contrast to a robust effect of devaluation evident in rats treated with vehicle during training (*F*(1, 16) = 11.05, *p* < 0.004). The difference between groups cannot be explained by a difference in consumption during the 1-hr pre-feeding phase as consumption of neither pellets (*t*(16) = 1.08, *p* = 0.30) nor sucrose solution (*t*(16) = 0.13, *p* = 0.90) differed between groups (Fig. 3D).

**Figure 4:**
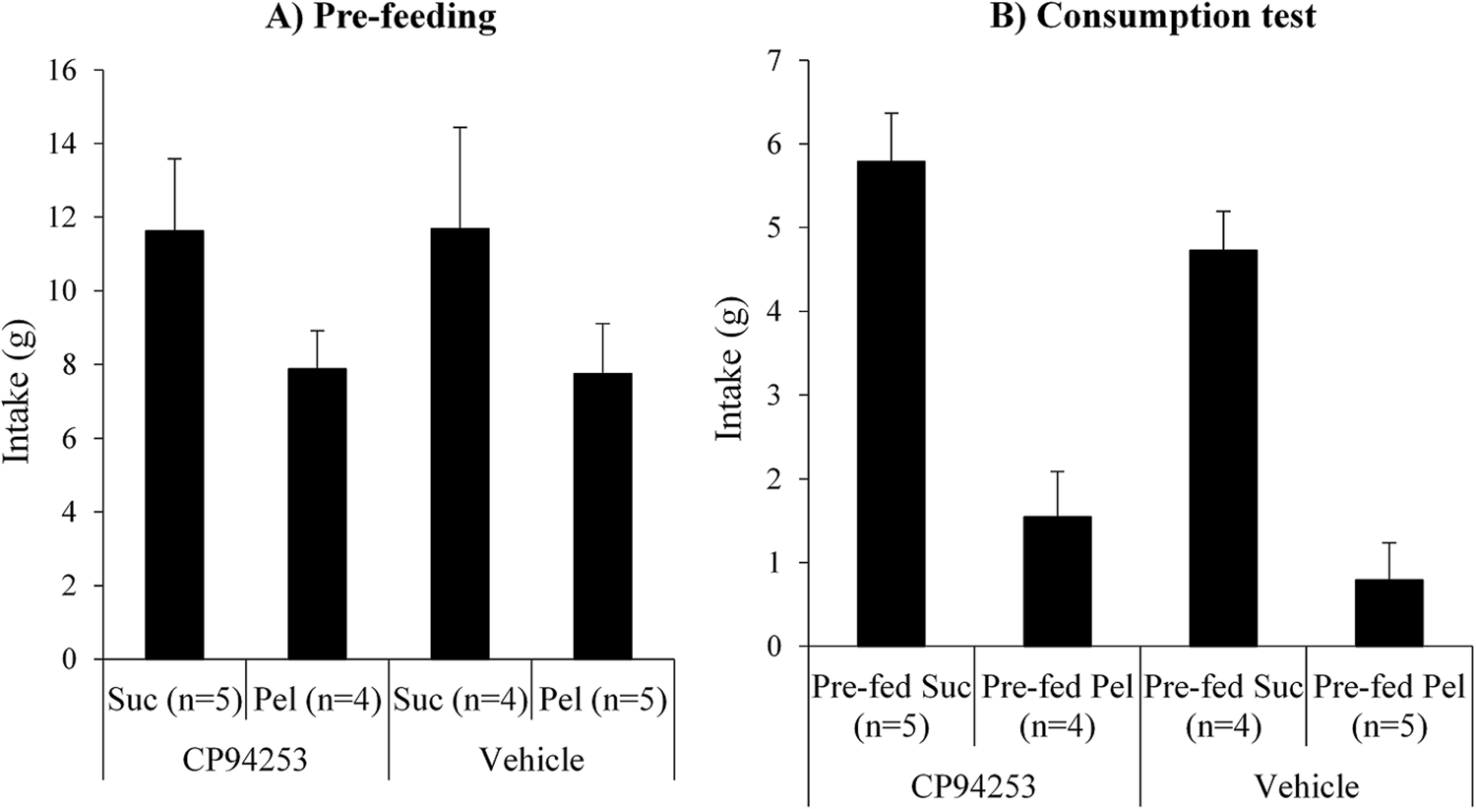
Devaluation consumption test. Animals were pre-fed either sucrose or pellets and then tested for further consumption of pellets. Animals in both CP94253 and vehicle groups reduced pellet consumption when pre-fed pellets compared to those pre-fed sucrose. Thus, the insensitivity of lever-press performance to the devaluation treatment was not due to compromised sensory-specific satiety produced by CP94253, since these rats, like the vehicle group, were sensitive to the effects of pre-feeding when measured in consumption.

Although consumption was similar between groups, it is possible that the resultant experience of satiety and, consequently, the efficacy of the devaluation treatment, differed between groups. Figure 4 shows the results from a follow-up consumption test held to confirm the impaired sensitivity to devaluation measured by instrumental responding was not due to ineffective satiety treatment. Thus, rats in CP94253 and vehicle groups were pre-fed either sucrose or pellets (*n* = 4 or 5 per group; Fig. 4A) for an hour – identical conditions to those preceding devaluation tests – prior to a 10-min test in which all rats were given a dish of pellets to eat (Fig. 4B). A 2 × 2 (group [CP94253 or vehicle] × pre-feeding [sucrose or pellets]) ANOVA found significantly lower pellet consumption in animals pre-fed pellets than those pre-fed sucrose (pre-feeding main effect: *F*(1, 14) = 61.89, *p* < 0.001) with no main effect of group (*F*(1, 14) = 3.05, *p* = 0.102) and no group × pre-feeding interaction (*F* < 1). Therefore, the insensitivity to devaluation shown by CP94253 rats in Fig. 3 was not attributable to a failure in sensory-specific satiety.

To explore the possibility that the lower overall responding in the drug group could have impacted our ability to detect a devaluation effect, we next examined sensitivity to devaluation in the lower- and higher-responding half of each group. After conducting a median split based on training response rates, we found the devaluation effect to be similar between the high- and low-responding subsets within each group. Thus, both high and low responding controls appeared sensitive to devaluation and both high and low responding CP94353 animals were not. (Supplemental Fig. S2).

### Reinforced test

**Figure 5:**
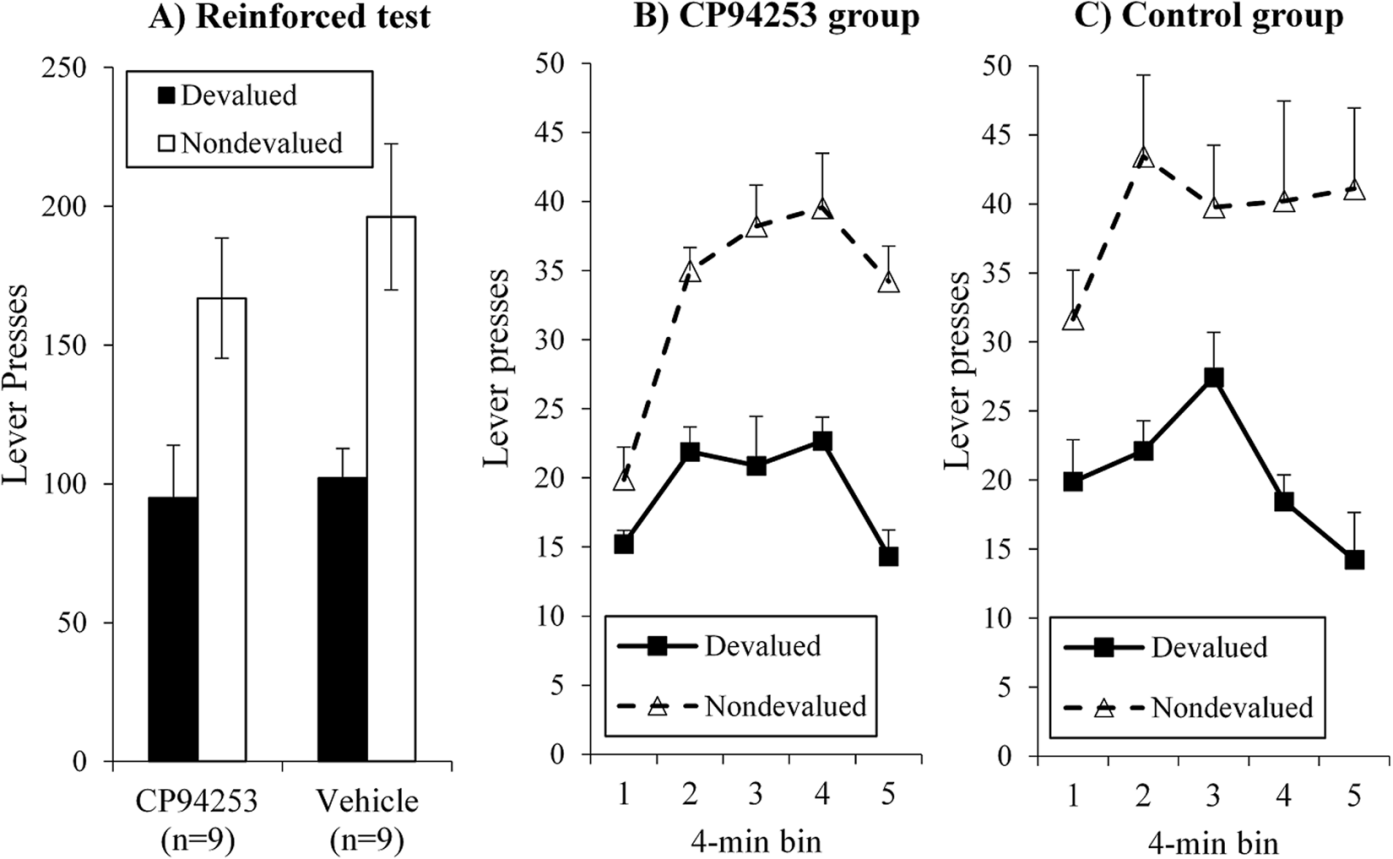
Reinforced test results. When lever pressing was reinforced by reward delivery after devaluation treatment, both groups selectively directed responding towards the lever earning the non-devalued outcome (Panel A). Analyses of data in 4-min bins showed this pattern was comparable for CP94253 (Panel B) and Control groups (Panel C).

A final test confirmed that, following pre-feeding, the deficit observed in CP94253 animals was rescued when lever-pressing in test conditions was reinforced by the delivery of the outcomes, as in training. These results are shown in Fig. 5, with total responding across a 20-min test shown in panel A and the same data presented in 4-min bins separated by group in panels B and C. Responding for pellets versus sucrose was collapsed because this factor did not alter the pattern of responding (no significant interaction or main effects when this factor was included in preliminary analyses). Data are instead organized according to whether the outcome earned by responding was currently devalued or non-devalued. Data were analysed in a 2 × [2] × [5] ANOVA (group × [lever] × [bin]), which found a significant overall devaluation effect (main effect of ‘lever’: *F*(1, 16) = 13.20, *p* = 0.002) that increased across time *F*(1, 16) = 4.25, *p* = 0.056), but critically, did not interact with group (lever x group interaction: *F* < 1). Overall responding during the test did not differ significantly between groups (*F*(1, 16) = 1.13, *p* = 0.30) and there were no other significant interaction effects.

## Discussion

Here we found that while peripheral administration of a selective 5-HT_1B_ receptor agonist (CP94253) allowed rats to acquire instrumental responding for food, when the content of that learning was further probed using an outcome devaluation task, performance differed from controls. CP94253 administered prior to learning about the unique outcomes associated with each lever resulted in animals being unable to selectively adjust responding to reflect the altered value of one, but not the other reward, in the test phase. The results indicate that 5-HT_1B_ agonism impairs learning about the specific sensory qualities of food rewards associated with distinct instrumental responses, required to direct choice performance when the value of one outcome has changed. The fact these deficits were detected when animals were tested drug-free is notable as it indicates that 5-HT_1B_ agonism altered the details of what was encoded during acquisition which then impacted responding even when animals were drug-free at test. This deficit cannot be explained by inability to discriminate between the different rewards or by the efficacy of the satiety treatment itself since both drug and vehicle groups showed a robust devaluation effect measured in consumption and ate comparable amounts of the outcomes during devaluation treatment itself (i.e., during induction of devaluation by specific-satiety). Further, in animals trained under 5-HT_1B_ agonism sensitivity to devaluation emerged when the outcomes were made available in a separate reinforced test. This suggests that they could use feedback from outcome delivery to direct responding appropriately and that their deficit was limited to choice responding based on an internal representation of the specific outcomes and/or action-outcome contingencies.

The hypothesis of this research was that a 5-HT_1B_ agonist would mimic the effects of increased *HTR1B* expression, as found to be associated with psychopathic personality traits in humans^13^, and would therefore be associated with diminished encoding of the specific features of outcome stimuli. This hypothesis was supported, as reflected by the apparent inability of the drug-treated animals to preferentially select the lever that lead to a non-devalued reward. As such, the serotonin 1B receptor is a plausible target for research into the cognitive substrates of psychopathic personality traits and the results support suggestions that the function of the serotonin system is altered in psychopathy.

In addition to associative learning deficits reported to be associated with PT, the amygdala has been found to differ in size^26^ and function^27,28^ in individuals with PT compared to those without and these differences may contribute to the etiology of PT. Furthermore, individuals with amygdala lesions show some similarities to people with PT such as difficulties in correctly recognizing others’ emotions and response-reversal deficits^4,29^. The altered amygdala function observed in individuals with PT together with what is known about the role of the amygdala in associative learning may yield insight into the observed deficits in learning tasks including devaluation. A recently developed model, the Differential Amygdala Activation Model (DAAM)^30^ argues that while there is substantial evidence to implicate amygdala function in PT, rather than treating the amygdala as a unitary structure, experimental findings are better explained by considering the distinct contributions of two important subregions of the amygdala; the central amygdala (CeA) and the basolateral amygdala (BLA). The model then draws from the animal associative learning literature to describe how understanding amygdala function could offer an explanation for the deficits in reinforcement learning shown by individuals with PT.

Lesion studies in animals have demonstrated that the CeA and BLA simultaneously and independently form associations between a stimulus or response and different aspects of the outcome; the CeA is involved with the encoding of the general motivational significance and valence of an outcome (good/bad) while the BLA is involved with encoding the specific sensory features of an outcome, linking those with the reinforcing properties of that outcome and updating that representation when value changes. As such, much like rats administered CP94253, while rats with lesions of the BLA acquire instrumental responding for food reward, they non-selectively reduce responding when one instrumental outcome is devalued^31,32^. The DAAM posits that in people with PT, the amygdala is differentially activated such that the BLA is chronically underactivated while the CeA is functioning normally; and this suggestion has recently gained empirical support from brain imaging studies^33,34^. Since associating stimuli or responses with specific features of reinforcing events relies on the BLA, altered function of this structure would result in a style of encoding that neglects the specific features (and particular value) of an outcome; information that is required in order to facilitate flexible and appropriate responding to associated stimuli or responses (e.g., to choose a course of action based on the value of different outcomes or to modify performance when expected value changes). Basic reinforcement processes, e.g., learning to perform a response for reward, should nonetheless be intact as this can be supported by a typically functioning CeA. There is some empirical evidence to support the suggestion that the reinforcement learning deficits seen in people with PT are linked to diminished encoding of the specific features of outcomes. In a learning task that was designed to put specific feature encoding in opposition with that of general motivational valence, a positive association was found between PT and general motivational valence encoding in both a sample of healthy adult males and a clinical sample of children with conduct problems^35^. In other words, people with PT had a style of encoding that neglected specific-features of outcomes. Behaviour that is inflexible and that does not respond appropriately to changes in the reinforcing properties of different outcomes may appear to be: risky (in the context of an increase in the probability of a negative outcome) or unkind (in the context of a dyadic interaction in which the behaviour causes a decreasingly positive response in the dyadic partner). In these situations, it is the continued engagement of a previously reinforced behaviour that gives rise to two of the personality features associated with PT. The DAAM hypothesizes that the chronic underactivation of the BLA is due, in part, to altered serotonin neurotransmission. For example, within the amygdala, a high density of serotonergic innervation has been found in the basal and lateral, but not central amygdaloid nuclei^20,36^. As such, within the amygdala, altered serotonergic transmission is likely to selectively alter BLA function and behaviours that rely on these nuclei. Indeed, localized infusions of serotonin selective reuptake inhibitors (SSRIs) into the BLA have behavioural effects, such as a reduction in the conditioned-fear response^37^. Together, there is substantial overlap between the symptoms of PT and the behavioural effects of BLA lesions; serotonin function within the BLA may link the two.

While the current data provide a useful proof of concept demonstration of the importance of 5-HT_1B_ receptors in outcome encoding, there are several limitations to the current study. Importantly, while 5-HT_1B_ agonism with CP94253 produces effects that are similar to those produced by BLA lesions, since this research used systemic administration of the agonist it is not known exactly which 5-HT_1B_ receptors were stimulated. Future studies administering CP94253 directly to the BLA would extend the current findings and more directly test the tenets of the DAAM model.

The CP94253-treated group responded less in training and consequently had less exposure to the two distinct outcomes which may have impacted their ability to form outcome-specific representations. Some findings suggest that response reinforcer associations may continue to grow with extended training^38^ and so stronger associations in the control group may contribute to a larger devaluation effect, although others have found that extended training, even under choice conditions, can favor habitual responding^39,40^. However, we think this is unlikely to fully explain the current results because previous studies employing a similar design but using more limited training (1–3 days), and thus reduced outcome exposure, have nonetheless generated robust devaluation effects^41–43^ indicating that the exposure in the drug group ought to have been sufficient to allow learning about the distinct outcomes, at least in intact animals. In fact, Colwill and Rescorla (1998)^38^ found that as few as 40 response-outcome pairings was sufficient to produce considerable response-reinforcer learning detectable with devaluation testing and so even if associations were stronger in control animals, CP94253-treated animals had enough training to allow specific associations to form yet they showed no indication of any selective sensitivity to the devaluation treatment. Future studies using yoked procedures to equate the number of response-outcome pairings across groups could more fully examine this issue.

It is possible that drug-induced satiety accounts for the lower instrumental response rates and reduced number of earned rewards during training as previous research has found that CP94253 promotes satiety^44,45^. While the choice of drug dose was based on previous reports that CP94253 in this dose range does not affect self-administration of sucrose or sweetened milk^22,23^ it is possible that motivation to respond for food reward was reduced in the drug group if these rats were experiencing drug-induced satiety. Nonetheless, we do not believe this potential effect of the drug can account for the test data of this experiment as no drug was given on the test day, and any lasting effects of the drug seem unlikely since consumption during the induction of specific-satiety was similar for the two groups. It is notable that devaluation in animals trained under CP94253 produced a nonselective decrease in responding at test rather than having no effect on performance. We believe that this is largely due to the method of devaluation. When animals are satiated prior to testing, control animals with intact representation of the specific features and value of each outcome are able to direct their responding for the still-valued outcome. However, if the feature-specific outcome representation needed to choose between the two responses is compromised, as we hypothesize for the CP94253 group, responding for reward in general may be indiscriminately diminished when animals are not hungry. Notably, this is the exact pattern seen following multiple demonstrations of impaired sensitivity to outcome devaluation following manipulation of the BLA, and indeed, in addition to indiscriminate responding, the overall rates of responding (<5 lever presses/minute) are similar^31,32,46–48^.

It has also been reported that CP94253 can have anxiogenic effects^25^. Indeed, this effect was utilized in this experiment as a positive control to provide evidence for sufficient drug dosage. As found previously, CP94253 appeared to have an anxiogenic effect on the animals in this experiment as drug-administered animals spent less time in the open arms of the elevated plus maze. There is no clear consensus on what the effect of an anxious state is on instrumental learning, but it should be considered as a potential confound. While any effects of anxiety on outcome encoding are largely untested, studies in both humans and animals suggest acute stress before, or during, testing can shift responding from goal-directed to habitual control^49–51^. For example, Pritchard *et al*. (2018)^49^ found that the induction of stress during the extinction test following specific satiety of one of two possible outcomes disrupts outcome devaluation while leaving reacquisition intact.

While the drug dosage used in this experiment induced anxiety in a test designed to bring out such effects, insensitivity to devaluation is unlikely to be a result of anxiety produced by the drug. First, while drug administration before training may have produced anxiety, since the drug was not administered prior to testing, the animals ought not to have been anxious at test. Any effects must then relate to how the drug affected what animals learned during training. Given that research suggests that stress affects instrumental behaviour primarily via processes involved in performance rather than acquisition^49–53^ it is unlikely that our insensitivity to devaluation effect was driven by anxiety states during acquisition. Second, evidence from the Posttraumatic Stress Disorder literature suggests that anxious states are associated with an increase of perceptual coding of stimuli^54^. It is the perceptual information (the features of the rewarding outcome) that seems lacking in the drug-administered rats and so even if rats were somewhat anxious during training this does not explain the pattern of results. Nonetheless, this finding is problematic for a model of PT. Typically, PT is characterized by low levels of anxiety. However, because 5-HT_1B_ receptors can act as either autoreceptors or heteroreceptors, it is possible that CP94253 may have different effects depending on what type and population of 5-HT1B receptors are affected following systemic administration. Future research may benefit from more localized manipulation of 5-HT_1B_ receptors that may be able to isolate effects of the drug on reinforcement learning from those on anxiety. Specifically, intra-BLA infusions of CP94253 would give a more accurate estimate of the role of 5-HT_1B_ terminal autoreceptors within the BLA on reinforcement learning. Alternatively, viral gene transfer could be used to target only the 5-HT1B receptors acting as autoreceptors to assess whether either the anxiogenic or the cognitive effects of CP94253 are linked with differences in receptor function. While such strategies have less translational appeal, they nonetheless could help isolate the locus of receptors essential to the clinical trait, and it is possible that in time these populations could be selectively targeted with less invasive techniques.

Finally, because the rats were trained under drug and tested drug free, we cannot rule out state-dependent learning effects. However, here our explicit aim was to manipulate 5-HT_1B_ function during the acquisition of two specific action-outcome associations to assess whether this altered the nature of reward encoding. Future studies specifically targeting BLA may mitigate any potential state dependent learning effects.

In summary, the DAAM model hypothesizes that hypofunction of the BLA resulting from increased expression of *HTR1B* leads to altered encoding of rewarding events; while basic reinforcement mechanisms are intact, individuals with PT lack detailed representations of specific outcomes and thus perform poorly when specific outcome values are changed, and representations require updating. We were able to mimic this effect with systemic administration of a 5-HT_1B_ receptor agonist, administration of which lead to a reduced sensitivity to outcome devaluation. Future research targeting the BLA could reduce the side effect profile and provide further support for the DAAM model. Learning that is insensitive to differences in the specific features of outcomes and changes in the value of some of those outcomes, results in behaviour that is inflexible and unreceptive. In dyadic contexts where the constant monitoring of, and sensitivity to social cues is critical, this could manifest as a lack of responsiveness to the changing behaviours and expressions of others. The individual would appear to be unempathic and emotionally indifferent; two of the hallmark features of psychopathy.

## Data Availability

The datasets generated during and/or analysed during the current study are available from the corresponding author on reasonable request.

## Supplementary information

Supplementary figures

## References

[1] Hare RD (1999). Psychopathy as a Risk Factor for Violence. Psychiatric Quarterly.

[2] Viding E, Fontaine NMG, McCrory EJ (2012). Antisocial behaviour in children with and without callous-unemotional traits. Journal of the Royal Society of Medicine.

[3] Budhani S, Blair RJ (2005). Response reversal and children with psychopathic tendencies: success is a function of salience of contingency change. Journal of child psychology and psychiatry, and allied disciplines.

[4] Mitchell DG (2006). Instrumental learning and relearning in individuals with psychopathy and in patients with lesions involving the amygdala or orbitofrontal cortex. Neuropsychology.

[5] Budhani S, Richell RA, Blair RJ (2006). Impaired reversal but intact acquisition: probabilistic response reversal deficits in adult individuals with psychopathy. J Abnorm Psychol.

[6] Mitchell DG, Colledge E, Leonard A, Blair RJ (2002). Risky decisions and response reversal: is there evidence of orbitofrontal cortex dysfunction in psychopathic individuals?. Neuropsychologia.

[7] Blair RJ (2005). Applying a cognitive neuroscience perspective to the disorder of psychopathy. Dev Psychopathol.

[8] Patrick CJ (1994). Emotion and psychopathy: Startling new insights. Psychophysiology.

[9] Scerbo A (1990). Reward dominance and passive avoidance learning in adolescent psychopaths. Journal of Abnormal Child Psychology.

[10] Blair KS, Morton J, Leonard A, Blair RJR (2006). Impaired decision-making on the basis of both reward and punishment information in individuals with psychopathy. Personality and Individual Differences.

[11] Dolan MC, Anderson IM (2003). The relationship between serotonergic function and the Psychopathy Checklist: Screening Version. Journal of psychopharmacology (Oxford, England).

[12] Glenn AL (2011). The other allele: exploring the long allele of the serotonin transporter gene as a potential risk factor for psychopathy: a review of the parallels in findings. Neurosci Biobehav Rev.

[13] Moul, C., Dobson-Stone, C., Brennan, J., Hawes, D. J. & Dadds, M. R. Serotonin 1B receptor gene (HTR1B) methylation as a risk factor for callous-unemotional traits in antisocial boys. *PLoS ONE* **10**, 10.1371/journal.pone.0126903 (2015). 10.1371/journal.pone.0126903 PMC4436296 25993020

[14] Moul C, Dobson-Stone C, Brennan J, Hawes D, Dadds M (2013). An exploration of the serotonin system in antisocial boys with high levels of callous-unemotional traits. PLoS ONE.

[15] Worbe Y, Savulich G, de Wit S, Fernandez-Egea E, Robbins TW (2015). Tryptophan Depletion Promotes Habitual over Goal-Directed Control of Appetitive Responding in Humans. International Journal of Neuropsychopharmacology.

[16] Worbe Y (2016). Valence-dependent influence of serotonin depletion on model-based choice strategy. Mol Psychiatry.

[17] Barnes NM, Sharp T (1999). A review of central 5-HT receptors and their function. Neuropharmacology.

[18] Duan J (2003). Polymorphisms in the 5’-untranslated region of the human serotonin receptor 1B (HTR1B) gene affect gene expression. Mol Psychiatry.

[19] Clark MS, Vincow ES, Sexton TJ, Neumaier JF (2004). Increased expression of 5-HT1B receptor in dorsal raphe nucleus decreases fear-potentiated startle in a stress dependent manner. Brain research.

[20] Hensler JG (2006). Serotonergic modulation of the limbic system. Neurosci Biobehav Rev.

[21] Hjorth S, Rui T (1991). The putative 5-HT1B receptor agonist CP-93,129 suppresses rat hippocampal 5-HT release *in vivo*: comparison with RU 24969. European Journal of Pharmacology.

[22] Przegalinski E, Golda A, Frankowska M, Zaniewska M, Filip M (2007). Effects of serotonin 5-HT1B receptor ligands on the cocaine- and food-maintained self-administration in rats. Eur J Pharmacol.

[23] Pentkowski NS, Acosta JI, Browning JR, Hamilton EC, Neisewander JL (2009). Stimulation of 5-HT(1B) receptors enhances cocaine reinforcement yet reduces cocaine-seeking behavior. Addiction biology.

[24] Pellow S, Chopin P, File SE, Briley M (1985). Validation of open:closed arm entries in an elevated plus-maze as a measure of anxiety in the rat. Journal of neuroscience methods.

[25] Lin D, Parsons LH (2002). Anxiogenic-like effect of serotonin(1B) receptor stimulation in the rat elevated plus-maze. Pharmacology, biochemistry, and behavior.

[26] Weber S, Habel U, Amunts K, Schneider F (2008). Structural brain abnormalities in psychopaths-a review. Behavioral sciences & the law.

[27] Schneider F (2000). Functional imaging of conditioned aversive emotional responses in antisocial personality disorder. Neuropsychobiology.

[28] Muller JL (2003). Abnormalities in emotion processing within cortical and subcortical regions in criminal psychopaths: evidence from a functional magnetic resonance imaging study using pictures with emotional content. Biol Psychiatry.

[29] Adolphs R, Baron-Cohen S, Tranel D (2002). Impaired recognition of social emotions following amygdala damage. Journal of cognitive neuroscience.

[30] Moul C, Killcross S, Dadds MR (2012). A model of differential amygdala activation in psychopathy. Psychological review.

[31] Balleine BW, Killcross AS, Dickinson A (2003). The effect of lesions of the basolateral amygdala on instrumental conditioning. The Journal of neuroscience: the official journal of the Society for Neuroscience.

[32] Corbit LH, Balleine BW (2005). Double dissociation of basolateral and central amygdala lesions on the general and outcome-specific forms of pavlovian-instrumental transfer. The Journal of neuroscience: the official journal of the Society for Neuroscience.

[33] Yoder KJ, Porges EC, Decety J (2015). Amygdala subnuclei connectivity in response to violence reveals unique influences of individual differences in psychopathic traits in a nonforensic sample. Human brain mapping.

[34] Aghajani, M. *et al*. Disorganized Amygdala Networks in Conduct-Disordered Juvenile Offenders With Callous-Unemotional Traits. *Biol Psychiatry*, 10.1016/j.biopsych.2016.05.017 (2016). 10.1016/j.biopsych.2016.05.017 27502216

[35] Moul C, Dadds MR (2013). Learning-style bias and the development of psychopathy. Journal of Personality Disorders.

[36] Smith HR, Porrino LJ (2008). The comparative distributions of the monoamine transporters in the rodent, monkey, and human amygdala. Brain structure & function.

[37] Kitaichi Y (2014). Local infusion of citalopram into the basolateral amygdala decreased conditioned fear of rats through increasing extracellular serotonin levels. Progress in neuro-psychopharmacology & biological psychiatry.

[38] Colwill RM, Rescorla RA (1988). The role of response-reinforcer associations increases throughout extended instrumental training. Animal Learning & Behavior.

[39] Killcross S, Coutureau E (2003). Coordination of actions and habits in the medial prefrontal cortex of rats. Cerebral cortex (New York, N.Y.: 1991).

[40] Gremel CM, Costa RM (2013). Orbitofrontal and striatal circuits dynamically encode the shift between goal-directed and habitual actions. Nature Communications.

[41] Yin HH, Knowlton BJ, Balleine BW (2005). Blockade of NMDA receptors in the dorsomedial striatum prevents action-outcome learning in instrumental conditioning. The European journal of neuroscience.

[42] Corbit LH, Janak PH (2010). Posterior dorsomedial striatum is critical for both selective instrumental and Pavlovian reward learning. The European journal of neuroscience.

[43] Corbit LH, Leung BK, Balleine BW (2013). The role of the amygdala-striatal pathway in the acquisition and performance of goal-directed instrumental actions. The Journal of neuroscience: the official journal of the Society for Neuroscience.

[44] Lee MD, Simansky KJ (1997). CP-94, 253: a selective serotonin1B (5-HT1B) agonist that promotes satiety. Psychopharmacology (Berl).

[45] Lee MD, Kennett GA, Dourish CT, Clifton PG (2002). 5-HT1B receptors modulate components of satiety in the rat: behavioural and pharmacological analyses of the selective serotonin1B agonist CP-94,253. Psychopharmacology (Berl).

[46] Parkes SL, Ferreira G, Coutureau E (2016). Acquisition of specific response-outcome associations requires NMDA receptor activation in the basolateral amygdala but not in the insular cortex. Neurobiology of learning and memory.

[47] Parkes SL, Balleine BW (2013). Incentive memory: evidence the basolateral amygdala encodes and the insular cortex retrieves outcome values to guide choice between goal-directed actions. The Journal of neuroscience: the official journal of the Society for Neuroscience.

[48] Johnson AW, Gallagher M, Holland PC (2009). The basolateral amygdala is critical to the expression of pavlovian and instrumental outcome-specific reinforcer devaluation effects. The Journal of neuroscience: the official journal of the Society for Neuroscience.

[49] Pritchard TL, Weidemann G, Hogarth L (2018). Negative emotional appraisal selectively disrupts retrieval of expected outcome values required for goal-directed instrumental choice. Cognition and Emotion.

[50] Fournier M, d’Arripe- Longueville F, Radel R (2017). Effects of psychosocial stress on the goal-directed and habit memory systems during learning and later execution. Psychoneuroendocrinology.

[51] Braun S, Hauber W (2013). Acute stressor effects on goal-directed action in rats. Learning & memory (Cold Spring Harbor, N.Y.).

[52] Schwabe L, Wolf OT (2009). Stress Prompts Habit Behavior in Humans. The Journal of Neuroscience.

[53] Schwabe L, Wolf OT (2010). Socially evaluated cold pressor stress after instrumental learning favors habits over goal-directed action. Psychoneuroendocrinology.

[54] Brewin CR, Dalgleish T, Joseph S (1996). A dual representation theory of posttraumatic stress disorder. Psychological review.

