# Supplementary material for: The role of serotonin 1B in the representation of outcomes
Source: Sci Rep. 2019 Feb 21;9:2497. doi: 10.1038/s41598-019-38938-4 (PMC6385264; doi:10.1038/s41598-019-38938-4)
Supplement: Supplementary file 1 — Supplementary figures [file 41598_2019_38938_MOESM1_ESM.docx]

**The role of serotonin 1B in the representation of outcomes**

Laura Corbit PhD^1,2^, Michael Kendig PhD^1,3^, Caroline Moul PhD^1*^

^1^School of Psychology, University of Sydney, Australia

^2^Department of Psychology, University of Toronto, Canada

^3^School of Medical Sciences, University of New South Wales, Australia

Corresponding author:

Caroline Moul

School of Psychology

University of Sydney

NSW

2006

Australia

Tel: +61 2 9036 6011

Email: caroline.moul@sydney.edu.au

**Supplementary Information**

*Figure S1.* Extinction test data were split by outcome. The top panel shows responding on the pellet lever when pellets had been devalued, or not. The bottom panel shows responding on the sucrose lever when sucrose had been devalued, or not. Responding overall was somewhat higher for pellets, but the effect of devaluation was very similar for the two outcomes.

*
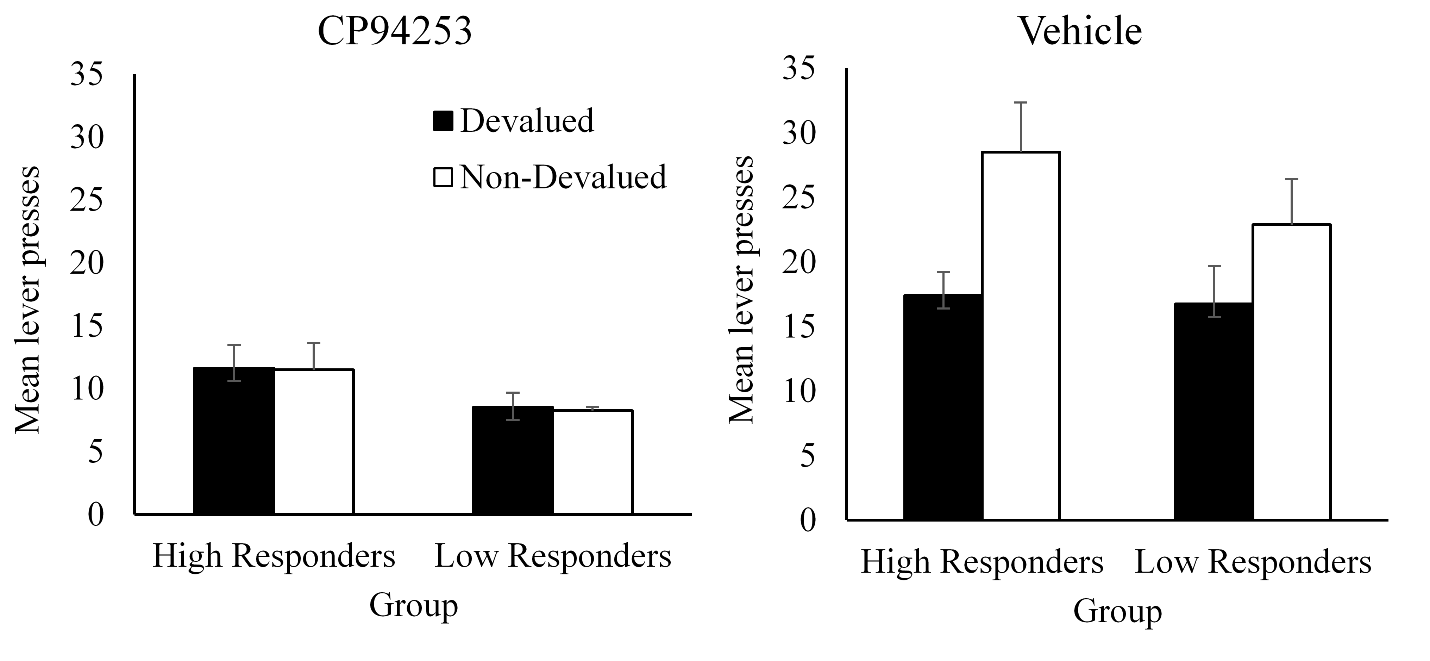
*

*Figure S2.* Sensitivity to devaluation was examined in the lower and higher responding halves of each group (n = 4 or 5 per group, based on response rates during RR5 training days 4 and 5). For controls, higher responders in training responded slightly more at test, but importantly there was no evidence that sensitivity to devaluation was different between higher and lower responders [no group by devaluation interaction: *F*(1,7)=0.365, *p* = 0.565]. The same pattern was seen in the drug group where overall responding varied according to training response rates, but sensitivity to devaluation did not differ between high and low responders (i.e., neither subset appeared sensitive to devaluation; no devaluation by group interaction; *F*(1,7) = 1.41, *p* = 0.273). This provides further evidence that sensitivity to devaluation, and particularly the lack of sensitivity to devaluation in the CP94353 group, was not an artefact of response rate.
